# A laparoscopic radical inguinal lymphadenectomy approach partly preserving great saphenous vein branches can benefit for patients with penile carcinoma

**DOI:** 10.1186/s12893-022-01582-3

**Published:** 2022-04-09

**Authors:** Yongkang Ma, Jianwei Hao, Huaqi Yin, Mingkai Zhu, Bao Guan, Chaoshuai Zhu, Bingqi Dong, Shiming Zhao, Zhaohong He, Tiejun Yang

**Affiliations:** 1 Department of Urology, Affiliated Cancer Hospital of Zhengzhou University, Henan Cancer Hospital, Zhengzhou, China; 2 Department of Urology, Henan Provincial People’s Hospital, Zhengzhou, Henan China

**Keywords:** Penile carcinoma, Inguinal lymphadenectomy, Laparoscope, Great saphenous vein branches

## Abstract

**Background:**

Inguinal lymphadenectomy (iLAD) is effective for penile carcinoma treatment, but usually results in many complications. This study aims to clinically evaluate the feasibility and clinical significance of a laparoscopic radical iLAD approach partly preserving great saphenous vein branches for penile carcinoma patients.

**Methods:**

A total of 48 patients with penile cancer who underwent laparoscopic radical iLAD with retention of the great saphenous vein in Henan Cancer Hospital from 2012 Jan to 2020 Dec were included in this study. Sixteen penile carcinoma patients who underwent laparoscopic radical iLAD preserving parts of superficial branches of the great saphenous vein were identified as the sparing group, and the matched 32 patients who incised those branches were identified as control group. This new procedure was performed by laparoscopy, preserving parts of superficial branches of the great saphenous vein, superficial lateral and medial femoral veins. Clinicopathological features and perioperative variables were recorded. Postoperative complications, including skin flap necrosis, lymphorrhagia, and lower extremity edema were analyzed retrospectively.

**Results:**

We found that the operative time of the sparing group is significantly longer than the control group (p = 0.011). There was no statistical difference in intraoperative blood loss, the lymph node number per side, average time to remove the drainage tube and postoperative hospital stay between the two groups. Compared to the control group, the sparing group showed a significantly decreased incidence of lower extremity edema (p = 0.018). The preservation of parts of superficial branches of the great saphenous vein was mainly decreased the incidence of edema below ankle (p = 0.034).

**Conclusions:**

This study demonstrated that the iLAD with preserving parts of superficial branches of the great saphenous vein, with a decreased incidence of postoperative complications, is a safe and feasible approach for penile cancer.

**Supplementary Information:**

The online version contains supplementary material available at 10.1186/s12893-022-01582-3.

## Introduction

The traditional procedure of inguinal lymphadenectomy (iLAD) for penile carcinoma is associated with a high incidence of complications, including skin flap necrosis and edema of lower extremities. In 2003, Bissoff [1] firstly applied the laparoscopic technology in iLAD for penile carcinoma, and confirmed that this procedure greatly reduced the risk of postoperative skin flap necrosis compared with traditional open surgery. Retention of the great saphenous vein during inguinal lymph node dissection under laparoscopy in penile carcinoma can reduce postoperative edema of lower limbs [2]. We wondered whether it would be feasible to preserve the great saphenous vein and its branches during iLAD procedure. Thus, we collected the perioperative variables and complications of penile cancer patients who were performed laparoscopic iLAD with retention of the great saphenous vein and assessed the clinical feasibility and therapeutic value of laparoscopic iLAD with retention of superficial branches of the great saphenous vein, superficial lateral and medial femoral veins.

## Materials and methods

### Clinical characteristics

**Fig. 1:**
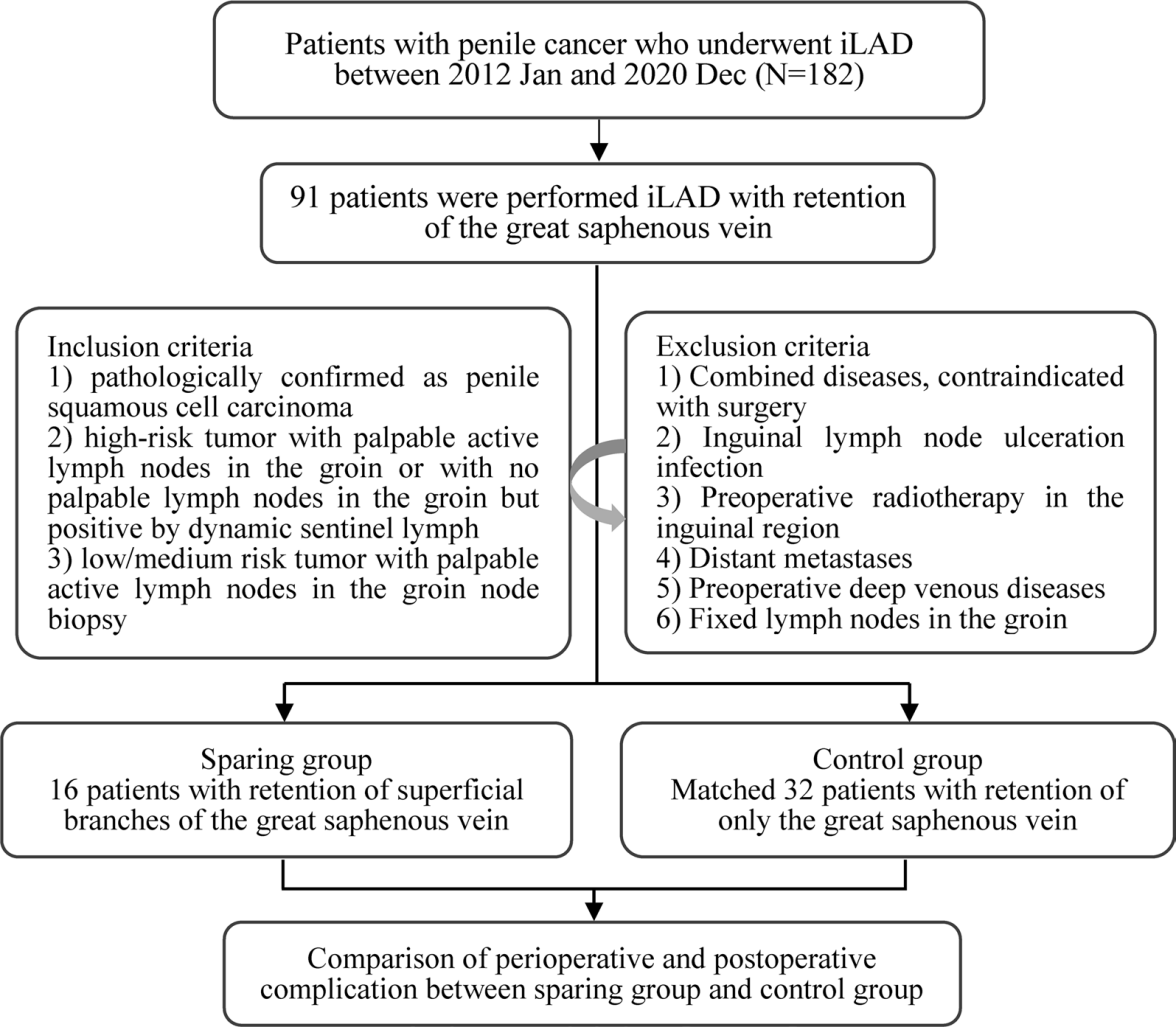
Workflow of patient selection

We retrospectively collected the records of 182 consecutive patients with penile carcinoma who received iLAD at Henan Cancer Hospital between 2012 Jan and 2020 Dec. Inclusion criteria: (1) Local lesions were pathologically confirmed as penile squamous cell carcinoma; (2) The local lesions were high-risk, with palpable active lymph nodes in the groin or with no palpable lymph nodes in the groin but positive by dynamic sentinel lymph node biopsy; (3) The local lesions were of medium and low risk, and there were palpable active lymph nodes in the groin. Lymph node metastasis was confirmed by inguinal lymph node resection biopsy or fine needle biopsy. Exclusion criteria: (1) Combined diseases of cardiovascular and cerebrovascular system or coagulation system, contraindicated with surgery; (2) Inguinal lymph node ulceration infection; (3) Preoperative radiotherapy in the inguinal region; (4) Distant metastases; (5) Preoperative ultrasound assessment of lower extremity blood vessels revealed deep venous diseases such as thrombosis and venous valve insufficiency. (6) Fixed lymph nodes in the groin. A total of 91 patients were performed laparoscopic iLAD with retention of the great saphenous vein, of which 16 patients underwent operation with retention of superficial branches of the great saphenous vein, superficial lateral and medial femoral veins (sparing group), and other 75 patients were included in control set. Then we matched 32 patients in a 1:2 ratio as control group according to clinicopathological features, including age, body mass index (BMI), smoking, American Society of Anesthesiologists (ASA) score, tumor stage, lymph node grading and history of pelvic lymph node dissections. In this study, only one senior surgeon (T.J. Y.) performed the vein preservation operation, and three senior surgeons (Z.H. H., T.J. Y. and W.J. H.) performed the laparoscopic iLAD with retention of the great saphenous vein. The flowchart of patient selection was showed in Fig. 1. After matching, the differences of preoperative characters between the sparing and control groups were acceptable (Table 1).

**Table 1:** Clinical characteristics of 48 patients

	Sparing group	Control group	P value
Number	16	32	–
Age, year	54.81 ± 6.585	54.44 ± 5.46	0.835
BMI, kg/m^2^	23.94 ± 3.64	24.25 ± 3.60	0.779
Smoking			
Present	5 (31.25%)	9 (28.13%)	1.000
Absent	11 (68.75%)	23 (71.87%)	
ASA score			
1	12 (75%)	23 (71.88%)	0.762
2	4 (25%)	8 (25%)	
3	0	1 (3.12%)	
Tumor stage			
T1	6 (37.5%)	11 (34.38%)	0.946
T2	8 (50%)	16 (50%)	
T3	2 (12.5%)	5 (15.62%)	
Lymph node			
N1	6 (37.5%)	13 (75%)	0.869
N2	9 (56.25%)	17 (75%)	
N3	1 (6.25%)	2 (75%)	
Pelvic lymph node dissections			
Absent	15 (93.75%)	30 (93.75%)	1.000
Present	1 (6.25%)	2 (6.25%)	

*BMI* body mass index, *ASA* American Society of Anesthesiologists

### Preoperative preparation and equipment

Enhanced CT or MRI and ultrasound were carried out to assess distant metastasis and the local lymph node status, and general condition and laboratory examination were performed to rule out surgical contraindications. The pathological grading and staging of penile biopsy or resection were performed based on UICC TNM grading system [3]. Local expanded resection, partial penectomy or penectomy were selected according to the pathological result and the tumor location referring European Association of Urology guidelines recommendation [4]. Pre-operative preparation included washing the perineum with 3% Benzalkonium chloride 3 days before the operation. All the iLAD procedures were performed under general anesthesia.

### Surgical procedure

**Fig. 2:**
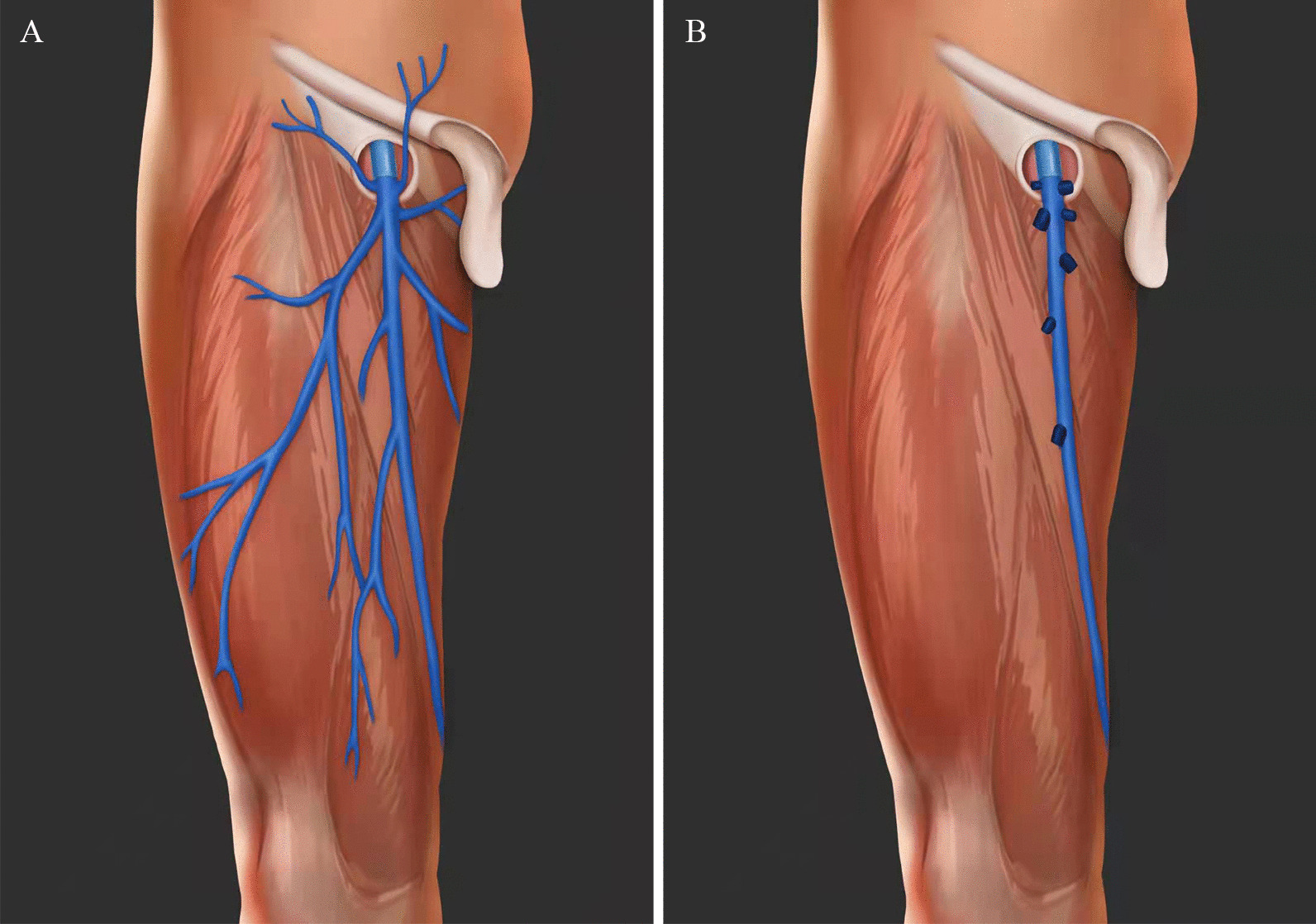
The mode picture showed the sparing group which preserving the great saphenous vein, its superficial branches, superficial lateral and medial femoral veins (**A**) and the control group which only retaining the great saphenous vein (**B**)

Patients were placed evenly in the supine position. The lower limbs were outreached at 30 degrees, and the knees bent at 120 degrees. One cm incision was first made 20 cm below the midpoint of the inguinal ligament. Skin was incised until the subcutaneous layer; the flap was separated bluntly from fat, with 12 cm half circle in direction to the groin, then inserting a 10 mm trocar for Surgical Cavity Mirror into the cavity. While connecting the pneumoperitoneum, the pressure was maintained at 10 mmHg. Second and third 1 cm incisions were made left and right to the first one, respectively, 5 cm close to the groin; 12 mm trocars were inserted into the cavity through the two cuts, respectively. Then, Separating the Camper’s fascia up to 3 cm above the inguinal ligament, the lateral margin to the Sartorius muscle, and the internal margin to adductor longus muscle. The great saphenous vein and its branches, superficial lateral- and medial- femoral veins were located, along the distal femoral triangle point. Then, vascular skeletonization of the great saphenous vein and its branches was performed by ultrasonic scalpel and self-made no damage rubber traction. Meanwhile, we tried to preserve the whole great saphenous vein, superficial medial and lateral femoral veins, unless they were too slim. The shallow level of superficial lymph nodes and fatty tissue located between fascia lata and Camper fascia was dissected. Along the 3 cm above the inguinal Canal level, the superficial epigastric vein was cut, and surrounding lymph nodes were harvested. All the tissues, such as adipose tissue and lymph nodes, between the flap and external oblique aponeurosis in this region were removed from top to the inguinal canal. The external pudendal vein was incised along the medial margin of adductor longus. The superficial iliac circumflex vein was cut along the lateral side of the sartorius muscle. The surrounding lymph nodes were removed along the inner side. Then, the other end of the superficial iliac circumflex vein, superficial epigastric vein, and external pudendal vein were cut at saphenous hiatus, close to the great saphenous vein. The femoral vascular sheath was opened in the femoral triangle. Then, vascular skeletonization of both femoral artery and femoral vein was performed. Lymph nodes surrounding deep femoral vessels were dissected. After the above steps, the tissues were removed in batches with a 12 mm Trocar. The surgical cavity was rinsed and soaked with sterile distilled water. Hemostasis was done perfectly. Drainage-tubes with negative pressure were inserted into the surgical cavity. Sometimes, refilling of the great saphenous vein could be observed. Wounds were sutured one by one. Elastic pressure bandages were used to fix flaps. After surgery, the superficial circumflex iliac vein, external pudendal vein, superficial epigastric vein surrounding lymph nodes (superficial lymph nodes), and femoral paraneoplastic oval fossa lymph node (deep lymph nodes) were separately sent for pathology. The trunk of the great saphenous vein was retained in control group, all branches were severed, and other steps were the same as sparing group (Fig. 2).

### Postoperative care and follow up

All patients received low-molecular weight heparin (85 IU/kg q12h by subcutaneous injection) for 5 days post-surgery. Elastic stockings were applied to prevent venous thrombosis of lower limbs. Dressing was changed every 3 days post-operation, and the flap color and subcutaneous fluid were observed carefully. Vascular ultrasound of lower extremities was carried out 5 days after surgery in order to assess deep venous thrombosis. The negative pressure drainage tube was removed with drainage volume below 10 ml in 24 h. The elastic bandage, however, was maintained for 1 more week. The observed intraoperative complications included operative time, blood loss, number of dissected lymph nodes, and time of drainage tube removal, and the postoperative complications includes poor flap healing, lower limb edema, lymphocysts, venous thrombosis.

All patients were followed in our department, their follow-up regimen included ultrasound, CT/MRI every 3 months within 2 years, and then were extended to 6 months thereafter.

### Statistical analysis

All statistical tests were performed with SPSS 22.0 (IBMCorp., Armonk, NY, USA), and two-side p < 0.05 was indicated as statistical significance. The chi-square test or Fisher’s exact test was used to determine the distribution of categorical variables, as indicated. The Student’s t tests were used for continuous variables.

## Results

In 48 surgical procedures, a total of 96 limbs were successfully completed. Patient ages were 54(range: 36–65 years) years. There was no significant difference in age, BMI, smoking status, ASA score and TNM staging between the sparing group and control group (Table 1). The mean operative time was 66.25 and 59.20 min/side in sparing group and control group, respectively (Table 2). The operative time of the sparing group is significantly longer than the control group (p = 0.011). There was no statistical difference in intraoperative blood loss, the lymph node number per side, time to remove the drainage tube and postoperative hospital stay between the two groups (Table 2).

**Table 2:** Perioperative information

	Sparing group	Control group	P value
Operative time (min/side)	66.25 ± 9.01	59.19 ± 8.38	**0.011**
Blood loss (ml/side)	19.69 ± 12.71	21.28 ± 11.29	0.660
Time for seal pressure drainage (day)	10.13 ± 4.47	11.03 ± 3.42	0.442
Postoperative hospital stay (day)	11.19 ± 5.00	11.28 ± 3.69	0.937
Clean the lymph node number (per side)	9.56 ± 1.90	9.78 ± 2.18	0.749

No skin flap necrosis and surgical intervention for flap healing was found in sparing group. Meanwhile, the incidence of subcutaneous infection incidence, lymphorrhagia, lymphocysts and venous thrombosis in sparing group and control group were 6.25% (1/16) and 9.37% (3/32), 18.75% (3/16) and 15.62% (5/32), 18.75% (3/16) and 18.75% (6/32), 12.5% (2/16) and 15.63% (5/32), respectively (Table 3). There was no significant difference in the incidence ofnecrosis of skin flap, subcutaneous infection, lymphorrhagia, surgical intervention for flap healing, lymphocysts and venous thrombosis. But edema of lower extremity incidence in sparing group is significantly lower than control group (p = 0.018). The preservation of parts of superficial branches of the great saphenous vein was mainly decreased the incidence of edema below ankle (p = 0.034) (Table 3).

**Table 3:** Postoperative complications

	Sparing group	Control group	P value
Necrosis of skin flap	0	2 (6.25%)	0.541
Subcutaneous infection	1 (6.25%)	3 (9.37%)	0.711
lymphorrhagia	3 (18.75%)	5 (15.62%)	0.654
Surgical intervention for flap healing	0	1 (3.13%)	1.000
Edema of lower extremity	1 (6.25%)	13 (40.62%)	**0.018**
Below ankle	1 (6.25%)	11 (34.37%)	**0.034**
Above ankle	0	2 (6.25%)	0.546
Lymphocysts	3 (18.75%)	6 (18.75%)	1.000
Venous thrombosis	2 (12.5%)	5 (15.63%)	0.772

Because of ≥ 2 inguinal lymph node metastases and enlarged pelvic lymph node, 3 patients underwent two pelvic lymph node dissections, with 1 patient displaying metastatic pelvic lymph nodes in control group (Table 1). After postoperative pathological findings of lymph node metastasis, the “ifosfamide + cisplatin + docetaxel” regimen was administered.

## Discussion

In traditional radical iLAD, the great saphenous vein and its branches should be cut, and the deep side and superficial inguinal lymph nodes are removed, transplanting tensor fascia lata to be used for covering femoral vessels. This procedure is the most effective treatment in penile carcinoma with lymph node metastasis. However, high incidence of complications, including skin flap infection, necrosis, edema of lower extremities, and lymphatic fistula, have been associated with this surgical method [5, 6]. With the development of laparoscopic techniques, many surgeons have successfully performed inguinal lymphadenectomy by laparoscopy. Endoscopic surgery can reduce complications with satisfactory tumor control [7]. Modified laparoscopic surgery such as preserving the great saphenous vein and fascia lata further decreases complications [2]. Catalona et al. [8] obtained a decrease in complications by retaining the great saphenous vein and reducing the sweeping range of downward and outside, but tumor control was worse than that of classic iLAD [9, 10]. Meanwhile, Zhou et al. [11, 12] reported that complications can be reduced dramatically by retaining fascia lata and transposing the sartorius muscle, although other sweeping areas were basically the same as the classic iLAD, and the validity of tumor control was not affected in this study.

There are five main great saphenous vein branches, including the superficial lateral femoral, superficial medial femoral, external pudendal, superficial epigastric, and superficial iliac circumflex veins. Lymphatic drainage flows from the lymph nodes surrounding the external pudendal vein to the perineal area close to the penis; meanwhile, studies have confirmed that part of the penis is affected by inguinal lymphatic drainage in the groin outside the area [13], as well as superficial epigastric vein and superficial iliac circumflex vein drainage areas. Therefore, we totally removed three branches as well as the surrounding tissues (Additional file 1).

The iLAD technique is crucial to clarify the patient’s pathological stage and assess survival [4]. In a retrospective analysis, iLAD complication rates reached 55.4%, and most complications were associated with surgical incision [14]. Application of laparoscopy in surgery changes this condition. There were fewer complications in comparison with conventional surgery. Our approach met the surgical scope of lymph node dissection. Besides, it was wider than Zhou’s surgery of the downward, with similar harvested lymph node counts [12]. In the early 90 s, Fraley [15] proposed to retain the great saphenous vein and its branches as much as possible in the open surgical method because by doing so postoperative complications can be reduced. In our study, we found that the surgery of retaining the great saphenous vein and its branches took more time, but it possessed an incomparable advantage that significantly reduced the incidence of lower extremity edema. In addition, for patients with abnormal status in deep veins of lower limbs before the operation, removing the great saphenous vein and its branches during iLAD gives rise to deep vein thrombosis of lower extremities or even a more serious neopathy. Such complications can be reduced by the surgical method preserving the great saphenous vein and its branches. In addition, the differences in postoperative complications can may be biased by the surgeons, but, in this study, three surgeons in this study were skilled in the surgery of retention of the great saphenous vein, and the clinicopathologic variables of the two group were matched to reduce the effect of confounding factors as far as possible. Consequently, we believe our finding could be validated by a large sample size study with a well study design.

This study has some shortcomings. The results of this study should be further validated by multicenter, a large scale of sample size cohort and long-term studies because of a retrospective nature. Another shortcoming of the current study fails to collect the information of patients’ outcome. Even so, we firmly identified that the new procedure descripted in this study could benefit to decrease postoperative complications.

## Conclusion

Endoscopic iLAD retaining the great saphenous vein and parts of its branches is a safe and feasible technique. This approach especially suits early penile carcinoma patients with suspicious lymph node metastasis, with a decreased incidence of postoperative complications. For penile carcinoma patients with deep vein disease of lower limbs, preserving the great saphenous vein and its branches may be a good option.

## Supplementary Information

**Additional file 1.** Clinical information of 48 penile cancer patients.

## Data Availability

The datasets in the current study are available from the corresponding author on reasonable request.

## Abbreviations

iLAD: Inguinal lymphadenectomy
BMI: Body mass index
ASA: American Society of Anesthesiologists
